# Replacing academic journals

**DOI:** 10.1098/rsos.230206

**Published:** 2023-07-19

**Authors:** Björn Brembs, Philippe Huneman, Felix Schönbrodt, Gustav Nilsonne, Toma Susi, Renke Siems, Pandelis Perakakis, Varvara Trachana, Lai Ma, Sara Rodriguez-Cuadrado

**Affiliations:** ^1^ Universität Regensburg, Regensburg, Germany; ^2^ IHPST, CNRS, Paris, France; ^3^ LMU, Munich, Germany; ^4^ Karolinska Institute, Stockholm, Sweden; ^5^ University of Vienna, Vienna, Austria; ^6^ Reutlingen, Germany; ^7^ Complutense University of Madrid, Madrid, Spain; ^8^ University of Thessaly, Larisa, Greece; ^9^ University College Dublin, Dublin, Ireland; ^10^ Autonomous University of Madrid, Madrid, Spain

**Keywords:** infrastructure, publishing, scholarly, replicability, affordability, functionality‌

## Abstract

Replacing traditional journals with a more modern solution is not a new idea. Here, we propose ways to overcome the social dilemma underlying the decades of inaction. Any solution needs to not only resolve the current problems but also be capable of preventing takeover by corporations: it needs to replace traditional journals with a decentralized, resilient, evolvable network that is interconnected by open standards and open-source norms under the governance of the scholarly community. It needs to replace the monopolies connected to journals with a genuine, functioning and well-regulated market. In this new market, substitutable service providers compete and innovate according to the conditions of the scholarly community, avoiding sustained vendor lock-in. Therefore, a standards body needs to form under the governance of the scholarly community to allow the development of open scholarly infrastructures servicing the entire research workflow. We propose a redirection of money from legacy publishers to the new network by funding bodies broadening their minimal infrastructure requirements at recipient institutions to include modern infrastructure components replacing and complementing journal functionalities. Such updated eligibility criteria by funding agencies would help realign the financial incentives for recipient institutions with public and scholarly interest.

## 1. Vicious cycle

After three decades of deterioration, more and more experts consider the scholarly journal system fundamentally broken and demand that it be replaced [1]. Most recently, Robert Terry, project manager at the World Health Organization stated at the R&I Days of the EC DG Research and Innovation that ‘The whole concept of a "journal" is kind of dead actually. What we need is a complete rethink', to strong support from the DG Jean-Eric Paquet [2] and reiterated by the Council of the EU [3]. Replacing traditional journals with a more modern solution is not a new idea [4–12], but the lack of progress since the first calls and ideas more than 20 years ago has convinced an increasing number of experts that the time for small tweaks is long gone and a disruptive break is now overdue.

The most prominent problem, with already realized legislative consequences, is the observation that empirical results can be less reliable than expected, an issue recognized as the *replication crisis*. Evidence suggests that the most prestigious journals, the ones that researchers must publish in or perish, are partly responsible for the observed lack of reliability by capitalizing on surprising, too-good-to-be-true results while lacking proper quality controls [13]. The journal system is financed by academic libraries who pay subscription and/or publication monies to an oligopoly of large international corporations who each own their separate monopoly not only on the scholarly content in their journals, but also on assigning academic credibility via their journal brands. Over several decades, their ‘single source exemption' from procurement rules has led to an *affordability crisis*: financial reports reveal corporate profit margins exceeding 40% and excess public spending of the order of a factor of ten, compared with market-based pricing [14]. A lack of digital modernization has caused a further *functionality crisis*, characterized not only by missing digital tools needed to combat unreliability, but also by researcher time wasted on antiquated procedures, e.g. in discovery, submission or review.

**Figure 1.**
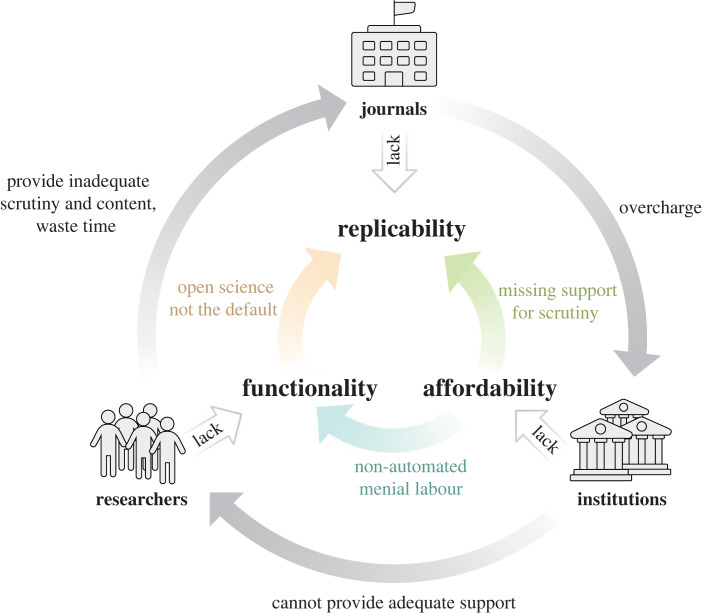
A vicious cycle of three crises. With their supra-inflationary price increases, profit-maximizing journals overcharge (via subscriptions or article processing charges) institutions by a factor of up to tenfold, extracting library budgets with little if anything left for infrastructural development. The resulting lack of infrastructure funds is a crisis of affordability: institutions cannot afford to invest in technology and its human support system that could relieve researchers of clerical tasks such as manuscript submission, data deposition, code publication etc. This results in a functionality crisis that entails researchers lacking time, functionalities and human support both for efficient scrutiny during the review process as well as for making their own research open and reproducible. Not shown: journals have apparently not invested their surplus into reviewer support, resulting in little improvement over the last decades, such that researchers are still lacking basic functionalities, such as e.g. comments via authoring system, direct author communication, AI-assisted error and fraud detection, efficient manuscript submission, etc. contributing to the functionality crisis. As the journals keep increasing their prices without a concomitant rise in investments, they fuel the replicability crisis.

These three crises fuel each other in a vicious cycle (figure 1): the affordability crisis prevents institutions from combating the functionality crisis. The functionality crisis, in turn, fuels the replication crisis, for instance by making peer-review more cumbersome and by making research data and code harder to discover, access, re-use and scrutinize. The journals propagating the replication crisis keep exacerbating the affordability crisis with super-inflationary price increases [14–16]. There are numerous other problems associated with current scholarly communication, but we consider these three to be the most pressing and that many of the others are mere consequences of the three mentioned here.

Thus, all three scholarly crises are interlocked in an ever-deteriorating vicious cycle, at the heart of which lies a public good in private hands: the scholarly literature. In this social dilemma, every player is at a disadvantage if they move (first), so they all remain locked-in: neither researchers—forced to publish in journals due to the ‘publish or perish' reality—nor libraries—serving the reading and publishing needs of their faculty—are in a position to initiate reform. The corporate publishers are the only player profiting from this system. They exploit this lucrative situation by using their massive profits not only to resist and delay any research- and public-oriented reform, but to fund a reform of their own and on their own terms. Their ‘reform' is not aimed at increasing the reliability of science or decreasing the financial burden on public institutions. Instead it aims to multiply corporate revenue streams and market power even further.

## 2. Surveillance capitalism

The major publishing houses, in no need of personalized advertisements due to handsome taxpayer funding, have recently been found to secretly track their academic users [17–21]. They use the data to create personal profiles of academic behaviour, such as when a user is searching for which topics, which journals or articles they open, or what sections of the documents they spend most time on. In their analysis, the German funding agency DFG has identified a whole host of unacceptable issues with this practice [17]. It constitutes a violation of fundamental rights: freedom of research and teaching are enshrined in the constitutions of many liberal democracies. Such tracking not only infringes on academic freedom, it also constitutes a violation of the right to protection of personal data, an encroachment of competition law, and reduces the value of public research investment, since data on research activities can be collected by commercial research competitors or be made available to them for a fee [17]. These are not merely abstract shortcomings, as tracking can expose scientists to specific and grave danger. From climate change to gender studies or racism: contentious areas of research are putting more and more scholars at risk. In Hungary, the government expelled the entire Central European University for political reasons, and in such cases, tracking information could be used to identify unruly academics to put pressure on them. As their behavioural profiles are tradable, they are open to all interested parties with the necessary funds. For instance, RELX, parent company of academic publisher Elsevier, is selling user data to the US Immigration and Customs Enforcement (ICE) [18].

## 3. Workflow monopoly

**Figure 2.**
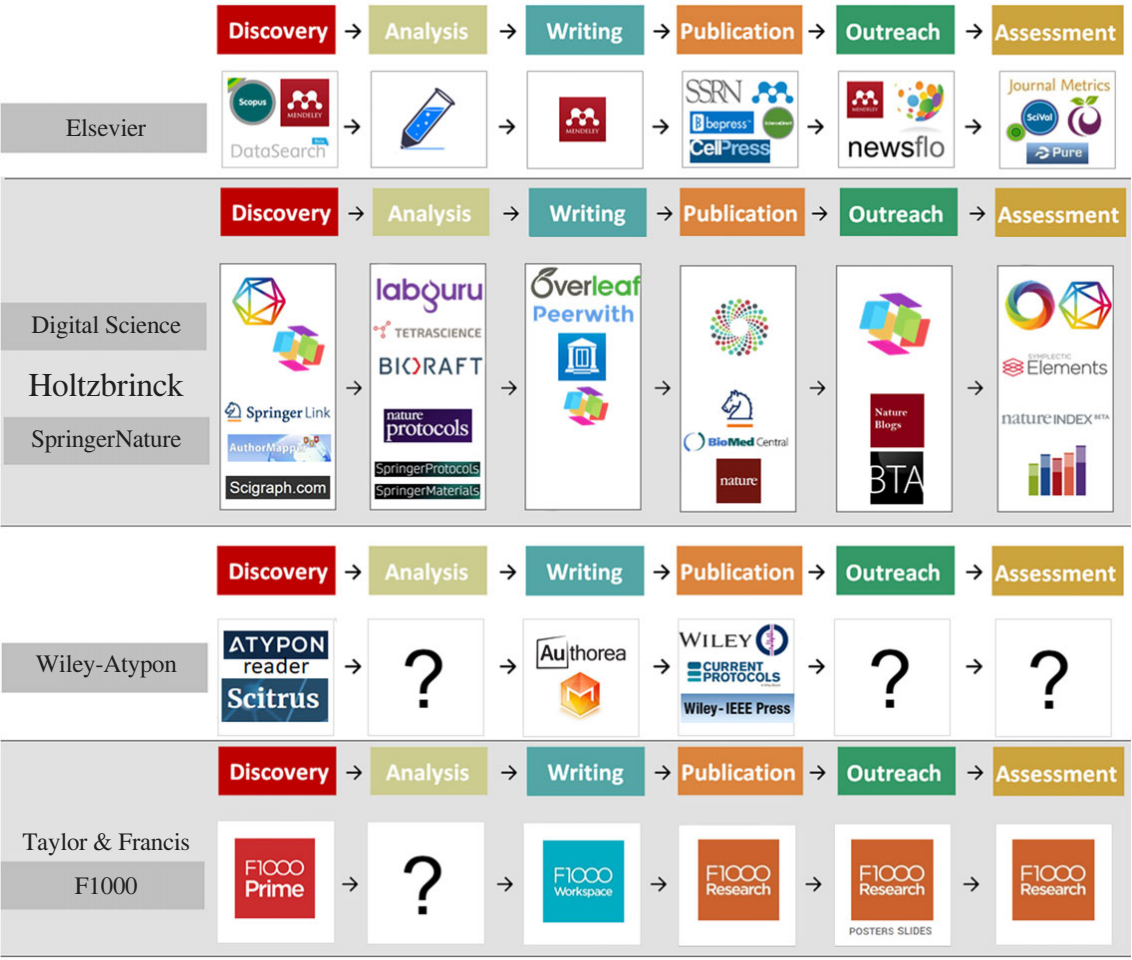
Providers of digital tools for the scientific workflow. Logos stand for software tools designed for specific aspects of the workflow. Each tool may be used in more than one step of the workflow. Elsevier and Holtzbrink are leading in the race to cover the entire workflow, with Holtzbrinck offering multiple tools for each step in the workflow. The preconditions for a functioning market exist, but a common standard is missing that provides for the substitutability of service providers or tools. (CC BY: Bianca Kramer, Jeroen Bosman, https://101innovations.wordpress.com/workflows.)

Additional revenue streams are only one of several reasons why so many academic publishing corporations deploy tracking technology [19–21]. Another is to expand their monopoly from research articles to research data and eventually the entire academic workflow. Over the last decade, the four leading publishers have all acquired or developed a range of services aiming to develop vertical integration over the entire scientific process from literature search to data acquisition, analysis, writing, publishing and outreach (figure 2). User profiles inform the corporations in real time on who is currently working on which problems and where. This information allows them to offer bespoke packaged workflow solutions to institutions. For any institution buying such a workflow package, the risk of vendor lock-in is very real: without any standards, it becomes technically and financially nearly impossible to substitute a chosen service provider with another one. In the best case, this non-substitutability will lead to a practically irreversible fragmentation of research objects and processes as long as a plurality of service providers would be maintained. In the worst case, it will lead to complete dependence of a single, dominant commercial provider. For academia, given the broad reach of such a monopolist, this worst-case scenario would constitute the equivalent of a Microsoft-SAP-Facebook monopoly. In the light of the experience with the scholarly literature, the expected consequences of such a development is that research quality, efficiency, and the free exchange of ideas and data could deteriorate substantially. The independence of universities and public research organizations—both national and EU—could drastically diminish. In that scenario, the current trifecta of crises might come to seem benign in comparison.

## 4. Replacement goal

The three aforementioned crises and the additional threat of a limited number of corporations gaining ownership of vast swathes of scientific data, including research and user data in addition to their share of the scientific literature, has led experts to call for a disruptive break from the current locked-in vicious cycle [2–10]. In particular, there is increasing agreement on the need to replace traditional journals with a modern scientific infrastructure: a decentralized (i.e. federated), resilient, evolvable network, based on open standards that allow seamlessly moving from one provider to another, under the governance of the scholarly community. Our companion publication [22] elaborates more on the value of federation. Most recently, the League of European Research Universities (LERU) emphasized that what is really needed ‘is the development of an open, inter-connected, publicly owned infrastructure' [23,24].

**Figure 3.**
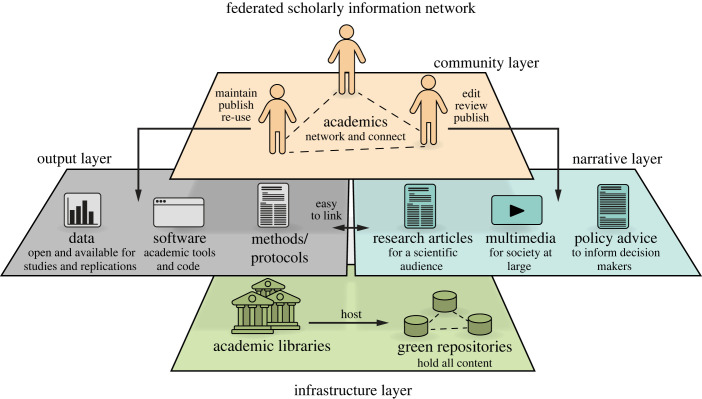
Concept for a federated scholarly information network. A federated network of institutional repositories constitutes the underlying infrastructure. Ideally, this infrastructure is designed redundantly, such that large fractions of nodes may go offline and the remaining nodes still provide 100% of the content. Users only directly interact with the output and narrative layers. The output layer contains all research objects, text, data and code. The narrative layer combines research objects in various forms, including research articles. The community layer encompasses standard social technologies such as likes, follows and other network tools (see also companion publication [22]). Modified from [25].

Because one agreed goal is to de-centre the journal article as the sole scientific output that ‘counts', the replacement needs to encompass all components of the scholarly workflow, with modern technologies taking care of text, data and code, allowing dynamic updating, version/quality control and tracking of contributorship (figure 3). It needs to replace the monopolies connected to the journals with a genuine, functioning and well-regulated market. In this new market, substitutable service providers compete and innovate according to the conditions of the scholarly community, avoiding vendor lock-in. The replacement should not only solve the current crises, but also prevent corporate capture of the scientific commons. To ensure the replacement is more resilient than the status quo, two minimal conditions have to be met. There needs to be a set of open standards according to which the technical infrastructure operates. There also needs to be a governing body that oversees and enforces implementation, development and resilience of the replacement. One could characterize the resulting federated scholarly information network with the term ‘resilient openness': open, but resilient against corporate capture and surveillance technologies.

## 5. Open standards

In order for workflow service providers to become substitutable, at a minimum, content and services need to be made accessible according to a set of enforced standards. To prevent commercial monopolization and to ensure cybersecurity, user/patient privacy and future development, these standards need to be open and governed by the scholarly community. Open standards enable switching from one provider to another, allowing public institutions to develop tender or bidding processes in which service providers can compete with each other to offer their services for the scientific workflow. A direct consequence of such standards is that the ‘single source exemption' from procurement rules—the reason for the power of legacy publishers—no longer applies. Freed from this exemption, the EU has established a standard tender process for their publishing platform Open Research Europe (ORE). Once that contract runs out, in contrast to the locked-in contracts with legacy publishers today, the EU may hold another tender for the subsequent contract(s). This process shows that it has now become realistic to aim for a journal system replacement that restores the regular procurement processes used in all other domains also to the digital scholarly infrastructure. There is no more need to reinvent the wheel for open scholarly infrastructures.

The basis for such standards exists [26–35] and only needs to be expanded (e.g. [36]), adopted and enforced. Such standards, evolving with feedback from the scientific community, would not only serve to make service providers interoperable, but they would also bring the means of scholarly communication back under the sovereignty of the scholarly community [37,38]. As an added benefit, the criteria defined in these standards (i.e. following Open Science and FAIR principles) not only allow for the substitutability of service providers but also assist scientists in following guidelines for good scientific practice. Such standards and the open infrastructures they enable thus prevent vendor lock-in, increase price pressure, promote innovation and increase the reliability of science. This replacement goal tackles the trifecta of scholarly crises all at the same time.

## 6. Governance

To govern standards equitably, a body analogous to e.g. the W3C needs to form under the governance of the scholarly community to allow the development of open scholarly infrastructures servicing the entire research workflow. A detailed proposal for the governance of such a body is beyond the scope of the present proposal, but the global work of UNESCO on the recommendations on Open Science and the stakeholder-driven governance of the Coalition for Advancing Research Assessment (CoARA) with a general assembly of all members and an elected Steering Board could provide inspiration. Vital is that the governing body or bodies would be assembled by competence and by representation of experts in this field, and that corporate capture is vigorously resisted (the W3C provides a warning example), which for example CoARA achieves by explicitly barring commercial actors from membership in the coalition (see also, e.g. [38,39]). Adequate, diverse representation complementing the competence of experts may be ensured in such a body/bodies by means of sortition. Drawing from experience of corporate capture both in centralized as well as distributed systems, the governance of the scholarly community ensures the practical implementation of ‘resilient openness' in the journal replacement.

## 7. Practical implementation

There is no shortage of technical solutions to implement a replacement, both from large service providers (figure 2) and from start-ups and community initiatives. More than 700 tools and solutions are ready to be implemented [40], once an open scholarly standard has been put into place. The technical feasibility of the basic infrastructure for preserving all digital research objects, including text, data and software code is not in question. What is very much in question, however, is to whom specifically to entrust the preservation of the world's scientific knowledge. The main candidates for this role are (i) public research/teaching institutions that already support the vast majority of researchers producing scientific knowledge, (ii) national or multinational consortia or governments (e.g. the EU) and their agencies, (iii) scholarly learned societies, or (iv) independent organizations.

Currently, solutions exist at all four levels. Scholarly institutions in particular have a long history of publishing the work of their scholars—be it in preprint archives or institutional repositories—and of striving to develop a global library of interoperable repositories [41–44], with CORE so far being the largest and most recent development [45]. Governments and funding agencies are also venturing towards such solutions, either by advancing policies designed to promote independence from monopolistic publishers [3,46] or by implementing their own solutions such as ORE from the EC see above. ORE is one node in the Open Research Central ORC network where some of the various other nodes are also represented by funding agency platforms. Humanities Commons HC is a social network built upon a cooperation of scholarly societies investing in a shared infrastructure. It was started by the Modern Language Association MLA which launched MLA Commons in 2013. Our companion publication [22] contains more details on the potential role of scholarly societies in a federated infrastructure. There are also independent, non-profit platforms where service providers can be substituted (e.g. Public Library of Science, PLoS; Open Library of the Humanities, OLH). While these still sport the journal container, they are demonstrating the feasibility of non-profit organizations overseeing scholarly infrastructures and developing revenue streams to keep the operations afloat. The newly established board of ORC is also now an independent body and hence fulfils the criterion of scholarly governance.

Thus it is clear that, taking the four levels together, all the components making up the various layers in figure 3 are already available in some form as a practical implementation today. Which option the scholarly community will choose will also set the path for what kind of standards are going to be adopted, depending on how the current implementations are operating. The choice is there, what is missing is an expert representation of the scholarly community to make these choices.

Once an alternative is established, every institution is empowered to replace their secret negotiations with regular tender processes, tackling the affordability crisis. Experience with tender processes is not limited to ORE. Scholarly societies have held tenders for their publication needs and the SCOAP3 project goes back over a decade [47–49]. These bidding processes commonly also specify the functionalities of the platforms, tackling the functionality crisis. When they are regulated to be fair and open, market forces can foster innovation, efficiency and benefit customers by exerting price pressure. This framework would create a completely new well-regulated market with genuine competition and eliminate the current monopoly conglomerate. Unlike the current system, this market will be easier to regulate, should dominant players arise. Moreover, the new framework needs not only to cater to traditional businesses but, being digital, will also encompass more modern forms of service providers on all scales between non-profit and for-profit. Open source software development has a decades-long tradition in the scholarly community and the new framework, by receiving funds currently supporting legacy journals, will finally offer sustainable support for it on a global scale (see below).

Infrastructure initiatives such as the European Open Science Cloud (EOSC) seamlessly integrate into the replacement framework due to the shared standards. The granularity of services and providers, package sizes and workflow solutions can be chosen by each institution and their users. Expert users are free to replace institutional components by components of their choice, or develop entirely new custom components themselves. Analogous to how institutions now can substitute one, say, electricity, heating or email provider with another one with minimal disruption, providers can in principle be substituted on any desired level of granularity; individual components or large package solutions. This disruptive break would bring the procurement rules of the digital scholarly infrastructure in line with those of the non-digital infrastructure.

## 8. Technical advantages

All scholarly content can be made available at single addresses despite the decentralized organization of the underlying, invisible, infrastructure. In that way, both global access to all scholarly content as well as access to sub-sections become conveniently available at any granularity. With the appropriate back-end solution, a large section (i.e. regions, countries, continents) of this decentralized network could go offline and the remaining nodes would still be able to offer 100% of the content [50]. In addition to this resilience, cybersecurity is increased even further by eliminating the tracking software currently deployed by publishers. Removal of the journal brand as a perverse incentive, together with automated data and code accessibility for scrutiny, tackles the two most important aspects of the replication crisis.

Dynamic updating and version control with persistent identifiers allows scholarly outputs to move from a static ‘version of record' to living outputs which can be rapidly updated to reflect the best available knowledge at any time (i.e. a ‘record of versions'). New models for tracking of contributorship and provenance will complement or even replace traditional authorship and help establish credit for valuable scientific activities not traditionally counted, such as generating and managing research data, peer review, brainstorming hypotheses or incremental improvements to open software (e.g. using the CRediT standard [27]). Improved peer-review functionalities such as direct author–reviewer interactions, peer-review aimed at all research output and not just narratives, transparent review, computer-assisted peer-review, and flexible anonymity and pseudonymity solutions contribute to more efficient quality control. Layered reputation systems for both research objects and users further support the trustworthiness of research objects and researchers. Visual interfaces, semantic search and content mining solutions not only save researchers time and improve the quality of the discovery process, but also allow software agents to derive novel hypotheses by analysing vast data networks with machine learning algorithms. All of these data and tools can fuel novel augmented intelligence systems, where artificial intelligence is combined with human intelligence to facilitate large-scale scientific collaboration.

Social technology will be built into the community layer. Increasingly adopted and supported by public institutions, including scholarly institutions, Mastodon is a social technology analogously federated as the journal replacement we propose here and hence well suited to be part of this new infrastructure. From Toots in Mastodon to print-on-demand monographs, a scholarly information network will cover scholarly communication in all its diverse forms. See our companion publication [22] for more on how to realize the social aspects of scholarship.

From improving essentially every single process in which scholars interact with their topic of study to machine learning-derived hypotheses, there are no downsides to replacing antiquated journals with modern technology. While any journal replacement will never be perfect, it will at least come with the tools and prerequisites to help mitigate researchers' all-too-human tendencies to cut corners, tell stories or cheat, in contrast to the journal system which has proven time and again to exacerbate these traits. The perfect should not be the enemy of the good.

## 9. Potential challenges

Some of the challenges already present today may not be immediately solved by replacing journals, but in many cases they stand to improve from the superior mitigation options such a replacement offers.

Clearly, the procurement rules in non-digital infrastructure are not a panacea. For instance, tender processes are not without problems and challenges [51,52]. Nevertheless, they provide sufficient advantages over other forms of public spending that they remain the dominant mechanism in most countries. Importantly, despite their problems, tender processes are superior to secret negotiations for transparency reasons alone. Moreover, once superior procurement methods improving on tenders have been developed, they can be applied to the journal replacement as well, marking yet another advantage.

Not all scholarly outputs can or should be made immediately and completely open and accessible. In disciplines and scenarios where privacy, patents, cultural sensitivities or proprietary data are an issue, embargoes or restricted access may have to be imposed. This is already possible in some institutional repositories and preprint archives. Importantly, in the current system, the default is closed science, with additional efforts required to make scholarship open. In the journal replacement, the default will be open and scholars get to decide how much effort they need to invest to safeguard their scholarship.

Market failure and corporate capture remain constant threats also for the new market envisaged here. The risks of so-called ‘free' markets under neoliberalism are well-known [53]. Therefore, the creation of functional market mechanisms is necessary to abolish the current monopolistic structures and prevent future vendor lock-in, but that alone is not sufficient. Proper regulation is essential, potentially involving introduction of first-sale doctrine in scholarly publishing [54], copyright reform [55–57], and most importantly, the monitoring of antitrust violations [58]. The journal replacement enables to leverage tried-and-tested market regulation mechanisms from other infrastructure domains such as electricity or telecommunications to be applied also for scholarly infrastructures to prevent monopolization of content and services. A straightforward example specific to scholarly works are overlay services that source the open content but reside outside of the network itself. Here, we can envision a wide range of services, both scholarly and commercial, designed to add value to research objects. This added value may include quality control through peer review, as in the case of Peer Community In, where a community of scholars reviews preprint articles on public servers independent of journals [59]. We can also imagine services running reproducibility checks and provide further quality assurance. This has already been implemented on the level of journals (e.g. the AEA data editor [60]), university services (e.g. *R*^2^ from Cornell University [61]) and independent ‘proof-testing' by so-called ‘Red Teams' [62]. It is easy to imagine how such existing services can acquire a dominant, quasi-monopolistic power. Once all scholarly content is accessible in a scholarly infrastructure, a whole host of new services stand to be developed. To prevent existing and new providers from exerting undue power, the scholarly community may decide, for instance, to prevent public funds from being spent on services that are not open source, increasing the chances of competitors arising or institutions to be able to offer the services themselves. A no-patent rule would be another measure. See also, e.g. [38,39,63].

A final challenge that needs to be addressed with respect to journal replacement is the reward structure currently baked into the monopolistic journal system. One of the core aims of this proposal is to defund the monopolists in order to remove the pernicious incentives they entail (see ‘Funding redirection' below). Removing pernicious incentives together with making the use of the journal replacement attractive and time-saving ought to be sufficient to change researcher behaviour. However, should this not be the case, the journal replacement offers a wealth of alternative quantitative and qualitative ways to empower selection or evaluation committees to make evidence-based choices without the current pernicious consequences. The main, but not exclusive factor here is the separation between how research is performed and communicated and the way it is evaluated—a central concept also emphasized by, e.g. DORA and CoARA.

## 10. Funding redirection

While a standards body under the governance of the scholarly community still remains to be formed, technically there do not seem to be any major hurdles for replacing journals. However, one crucial question remains that has not yet been settled, and it is not a technical, but a social or political question: with everybody locked-in, who is to act in which way to ensure the redirection of funds from the legacy system to the replacement solution, i.e. an open scholarly infrastructure?

Historically, funding agencies have ensured minimum working conditions at funded institutions by requiring specific infrastructures. Some funding agencies have expanded these eligibility criteria to also include criteria for good scientific practice, such as the German DFG [64]. Along the same veins, funding agencies have recently started to also include institutions' evaluation procedures in their eligibility criteria. Wellcome or Templeton World Charity Foundation are leading the way by refusing to fund applicants at institutions that evaluate researchers by the journals they publish in [65,66]. One does not need to completely exclude institutions from funding to incentivize change. For instance, in Finland's national funding model for universities, certain openly accessible research objects rank 20% higher than legacy objects [67]. With open research practices becoming more and more commonplace [68], such and analogous incentives for institutions to provide an infrastructure that not only supports these practices across the board but simultaneously addresses the most pressing scholarly problems is just common sense. Updating such funder guidelines and criteria to reward the redirection of recurrent funds towards open infrastructure components that tackle the three crises and, at the same time, disincentivize maintenance of funding for the legacy infrastructures that are fuelling the crises, would help realign the financial incentives for institutions with public and scholarly interest. With journal articles currently being overpaid by a factor of 10, the level of library funding is more than sufficient to implement any desired technology.

The European Commission, long having recognized the non-substitutability of academic publishers (‘the Commission found that consumers will rarely substitute one publication for another following a change in their relative prices' [69]), can be identified as another important actor. Regulatory bodies such as those in the EU may update their regulatory frameworks to include alternative scholarly publishing solutions such as those outlined above and declare that the traditional ‘single source exemption’ can no longer be applied by public scholarly institutions. As a consequence, the traditional institutional and consortial (and typically secret) negotiations with publishers would have to be scrapped and institutions would need to host tenders for their publishing needs. Such tenders exert real price pressure on bidding organizations and hence can free funds for the implementation of the funder requirements outlined above. The latest conclusions from the Council of the European Union support this perspective and call for solutions that are perfectly aligned with our proposals here [70].

Ownership involves socially recognized economic rights, first and foremost the exclusive control over that property [71], with the self-efficacy it affords. The inability to exert such control over crucial components of their scholarly infrastructure in the face of a generally recognized need for action for over three decades now, evinces the dramatic erosion of real ownership rights for the scholarly community over said infrastructure. Thus, this proposal is motivated not only by the now very urgent need to restore such ownership to the scholarly community, but also by the understanding that through funding bodies, scholars may have an effective and proven ally at their disposal to identify game-changing actions and to design a financial incentive structure for recipient institutions that can help realize the restoration of ownership, with the goal to implement open digital infrastructures that are as effective and as invisible as any non-digital infrastructure.

## 11. Summary

In summary, we argue that the time is ripe to replace traditional and outdated academic journals with an open, interoperable and community-governed modern scholarly information infrastructure. To this end, we recommend the following steps to be urgently taken:

— An international body (comprising not-for-profit organizations) should be formed to codify, develop and oversee implementation of open standards for a modern scholarly information system.
— Open, interoperable and publicly owned solutions for each facet and layer of scholarly information need to be extended, refined, sustained and, where necessary, created.
— Funding that is currently spent on journal publishing should be increasingly redirected towards supporting the new infrastructure. We identify two factors that can incentivize such re-allocation of funds:
  – Public research funders including governments should update the eligibility conditions for recipient institutions of their grants to include such infrastructure requirements.
  – Market regulators should enforce tenders as the method of procurement for scholarly communications infrastructures, preventing negotiations with monopolistic corporations.

The increasing momentum on reforming the criteria used for assessing research to focus on the intrinsic merits and openness of scholarly work instead of the prestige of the outlet where it appears makes this a uniquely opportune moment for a renewed push to modernize our scholarly information architecture. Similar to the case of research assessment itself, ultimately the choice—and the responsibility—of how we communicate research is in the hands of the scholarly community, if we only are able to seize the chance.

During the peer review process of this article, the Council of the EU has adopted conclusions on scholarly publishing, that echo our proposal here, e.g. ‘[The Council] encourages Member States and the Commission to invest in and foster interoperable, not-for-profit infrastructures for publishing based on open source software and open standards, in order to avoid the lock-in of services as well as proprietary systems, and to connect these infrastructures to the EOSC' [3]. On the same day, 10 major research organizations came out in support of the Council document [46]. We take this as a strong endorsement of the ideas in this and our companion article [22].

## Data Availability

This article has no additional data.
